# Dorsolateral Prefrontal Functional Connectivity Predicts Working Memory Training Gains

**DOI:** 10.3389/fnagi.2021.592261

**Published:** 2021-03-01

**Authors:** Sofia Faraza, Julia Waldenmaier, Martin Dyrba, Dominik Wolf, Florian U. Fischer, Kristel Knaepen, Bianca Kollmann, Oliver Tüscher, Harald Binder, Andreas Mierau, David Riedel, Andreas Fellgiebel, Stefan Teipel

**Affiliations:** ^1^Department of Psychosomatic Medicine and Psychotherapy, Rostock University Medical Center, Rostock, Germany; ^2^German Center for Neurodegenerative Diseases (DZNE), Rostock, Germany; ^3^Department of Psychiatry and Psychotherapy, University Medical Center Mainz, Mainz, Germany; ^4^Center for Mental Health in Old Age, Mainz, Germany; ^5^Institute of Movement and Neurosciences, German Sport University Cologne, Cologne, Germany; ^6^Leibnitz Institute for Resilience Research (LIR), Mainz, Germany; ^7^Institute of Medical Biometry and Statistics (IMBI), Faculty of Medicine and Medical Center, University of Freiburg, Freiburg, Germany; ^8^Department of Exercise and Sport Science, LUNEX International University of Health, Exercise and Sports, Differdange, Luxembourg

**Keywords:** functional connectivity, dorsolateral prefrontal cortex, working memory gains, cognitive training, healthy older adults

## Abstract

**Background:** Normal aging is associated with working memory decline. A decrease in working memory performance is associated with age-related changes in functional activation patterns in the dorsolateral prefrontal cortex (DLPFC). Cognitive training can improve cognitive performance in healthy older adults. We implemented a cognitive training study to assess determinants of generalization of training gains to untrained tasks, a key indicator for the effectiveness of cognitive training. We aimed to investigate the association of resting-state functional connectivity (FC) of DLPFC with working memory performance improvement and cognitive gains after the training.

**Method:** A sample of 60 healthy older adults (mean age: 68 years) underwent a 4-week neuropsychological training, entailing a working memory task. Baseline resting-state functional MRI (rs-fMRI) images were acquired in order to investigate the FC of DLPFC. To evaluate training effects, participants underwent a neuropsychological assessment before and after the training. A second follow-up assessment was applied 12 weeks after the training. We used cognitive scores of digit span backward and visual block span backward tasks representing working memory function. The training group was divided into subjects who had and who did not have training gains, which was defined as a higher improvement in working memory tasks than the control group (*N* = 19).

**Results:** A high FC of DLPFC of the right hemisphere was significantly associated with training gains and performance improvement in the visuospatial task. The maintenance of cognitive gains was restricted to the time period directly after the training. The training group showed performance improvement in the digit span backward task.

**Conclusion:** Functional activation patterns of the DLPFC were associated with the degree of working memory training gains and visuospatial performance improvement. Although improvement through cognitive training and acquisition of training gains are possible in aging, they remain limited.

## Introduction

The rapid increase in aging people among the population and age-related cognitive decline increase the need for interventions to maintain cognitive function, such as cognitive training. Previous findings demonstrated that cognitive training interventions can improve the performance of healthy older adults in cognitive tasks (Mahncke et al., 2006; Schmiedek et al., 2010; Kelly et al., 2014). A training is considered effective when the trained skills can be transferred to untrained tasks outside the training context in order to improve everyday cognitive functioning (Schneider, 2008; Strenziok et al., 2014). In addition, the maintenance of training gains is an important goal of cognitive training. Evidence of transfer of the training effect is mostly reported for untrained tasks within the same cognitive domain (near transfer effect), but rarely occurs across different domains (far transfer effect) (Blume et al., 2010; Kelly et al., 2014).

Although the capability of generalization of training gains to untrained tasks decreases with age, there is evidence that it remains possible in aging (Schmiedek et al., 2010; Wolf et al., 2014; Heinzel et al., 2016), and gains are maintained up to 18 months after the training in older people (Dahlin et al., 2008). This underlines the importance of exploring and understanding the neural mechanisms of transfer effects (Schmiedek et al., 2010).

Normal aging is associated with the structural and functional brain changes that can affect cognitive domains including working memory (Bopp and Verhaeghen, 2005). There are also studies demonstrating that cognitive deteriorations are more likely linked to alterations in the synaptic connectivity rather than to the frank neuronal loss (Morrison and Baxter, 2012; Bamidis et al., 2014). For example, synaptic changes during aging have been observed in the dorsolateral prefrontal cortex (DLPFC), and they could be related to working memory decline in healthy older adults (Rypma and D'esposito, 2000; Morrison and Baxter, 2012). The DLPFC is involved in cognitive processes, such as the maintenance and retrieval of information, and there is evidence that an electrical stimulation of this area leads to an increased quantity of information retrieved from the memory (Gray et al., 2015).

Findings from fMRI studies (Nagel et al., 2009; Heinzel et al., 2014) indicate an association between decreased working memory performance and age-related changes in functional activation patterns during task performance. Toepper et al. (2014) have used a spatial working memory task (Corsi block-tapping test) and conducted group comparisons between old and young participants and among old high-performers and old low-performers to assess differences in the brain activation and functional connectivity (FC) during spatial working memory retrieval. FC is defined as the coactivation of spatially segregated brain regions (Van Den Heuvel and Hulshoff Pol, 2010). Their results revealed that the old high-performers demonstrated higher FC of the right dorsolateral and anterior prefrontal cortex than the old low-performers. Moreover, a study from Steffener et al. (2012) revealed that a poor performance on a verbal working memory task was associated with changes in the FC between the brain networks. According to Cao et al. (2016), a high level of resting-state FC of bilateral DLPFC can be induced by multidomain cognitive training in comparison with the untrained group where the FC was decreased. In addition, several studies suggest an association between increased resting-state FC within cognitive networks after cognitive training, resulting in a higher level of cognitive performance (Langer et al., 2013; Chapman et al., 2015; Cao et al., 2016).

In the present study, we investigated the hypothesis that a multidomain cognitive training improves the performance of untrained working memory tasks, and the maintenance of training gains directly after the training and after a delay of 3 months is possible for older adults. Moreover, we aimed at investigating the association of FC of DLPFC during resting state with working memory performance (digit span and visual span backward retrieval) before and after cognitive training in healthy older adults. Following the evidence of previous studies (Chapman et al., 2015; Cao et al., 2016), we hypothesized that a high FC of DLPFC at baseline would predict the successful maintenance of training gains and the improvement of cognitive performance after the cognitive training beyond the trained task. In this study the control group was used as reference in order to examine the training gains and the differences between the two groups regarding the training effectiveness on cognitive performance; therefore, it was excluded from functional imaging. For the current analysis, we selected working memory *a priori* for two reasons. First, it is a very important cognitive domain contributing to many other cognitive processes, and second, we could identify well-established seeds to conduct a hypothesis-driven analysis on FC networks predicting change in working memory performance after training. By examining the association of baseline resting-state FC with training gains and working memory performance, we aimed to investigate the brain mechanisms of healthy older adults who could most benefit from the cognitive training. Most of the studies focus on the effects of cognitive training on functional activation of the brain (Takeuchi et al., 2013; Chapman et al., 2015; Cao et al., 2016) and on the predictive role of factors, such as baseline cognitive performance or physical activity, on cognitive training gains (Zinke et al., 2014; Rahe et al., 2015). To the best of our knowledge, the functional brain mechanisms as predictors of training gains and cognitive performance in healthy older adults have not been reported. Knowledge on these functional brain mechanisms is essential for the design of cognitive interventions.

Additionally, we examined the role of demographic characteristics, such as age, on the prediction of performance improvement on untrained working memory tasks and transfer of gains. Recent findings (Bürki et al., 2014; Zinke et al., 2014) revealed that younger age was associated with larger training gains and transfer effects after a working memory training. Moreover, following the findings from Zinke et al. (2014), we investigated if a poor baseline performance could also be a significant predictor of training gains.

## Materials and Methods

The following procedures, data analyses and results are a part of a larger longitudinal, interventional, parallel-group, multicenter, and multimodal-imaging trial named “AgeGain” focusing on effective cognitive and physical training to support cognitive functioning in aging (German Clinical Trials Register, ID: DRKS00013077). For more details about this clinical trial, see the study protocol of Wolf et al. (2018).

### Subjects and Procedure

In the present study, our sample consisted of 79 healthy older adults (48 female, mean age 68 years, SD: 6.59, range: 60–88) (see Table 2 on demographic characteristics). All participants were recruited by local newspaper announcements and flyers and they were enrolled by two recruiting centers in Germany: Mainz (University Medical Center Mainz—Department of Psychiatry and Psychotherapy) and Rostock [University Medical Center Rostock—Clinic of Psychosomatic and Psychotherapeutic Medicine and German Center for Neurodegenerative Diseases (DZNE)]. The inclusion criteria were age ≥60 years, sufficient knowledge of German language, ability to understand the content and consequences of a clinical trial, and sufficient mobility and motivation in order to take part in the examinations. Subjects were not included if they had current-or history of- psychiatric, neurological, cerebrovascular, or cognitive illness, brain lesions, if they were taking medications that could influence the cognitive performance, such as current use of medication for hormone replacement therapy for women or if there was any criterion that could affect MRI acquisition (such as metal or cochlear implants, tattoos, or pacemaker). Assignment to training and control group was based on random assignment and was stratified by the trial coordinator at the trial site University Medical Center Mainz. The participants that were in the experimental group (*N* = 60) got an expense allowance of 150 euros and those who were in the control group (*N* = 19) an allowance of 50 euros. The study was granted ethical approval by the local ethics committees of both trial sites: Mainz: Ethics Commission of the Landesärtzekammer Rheinland-Pfalz, Rostock: Ethics Commission of the Rostock University's Faculty of Medicine [Reference number: 837.385.15 (10153)]. All subjects provided written informed consent to participate in the trial.

**Figure 1:**
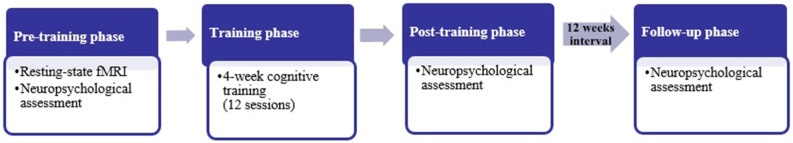
Illustration of the study design.

After the screening phase (inclusion/exclusion), the experimental group underwent MRI. Both groups (experimental and control) underwent a baseline neuropsychological assessment of ~4 h (pre-cognitive training phase). The cognitive training phase consisted of 12 sessions (1.5 h/session) over a 4-week period with 3 sessions per week. After 4 weeks of cognitive training, both groups repeated the neuropsychological assessment (post-cognitive training phase) to determine the short-term maintenance of training gains. Finally, after 12 weeks the neuropsychological assessment was again applied to both groups to assess the long-term maintenance of training gains (follow-up phase) (Figure 1). To avoid the memory effect on transfer measures, different versions of the same tests have been used in every neuropsychological assessment. After the completion of the study, all the participants received feedback about their cognitive performance. More specifically, they were informed about their progress on every task across the 4 weeks of cognitive training and about their performance in pre-training and follow-up neuropsychological assessments.

### Neuropsychological Materials

#### Cognitive Training

Cognitive training entailed computer-based cognitive tasks that cover a wide range of cognitive domains and contribute to improving cognitive functions. The selection of cognitive tests was theory-driven and based on empirical findings. More specifically, for working memory training, we used the computerized training software Training and Testing Tool (TATOOL, Java-based open-source programme available at www.tatool.ch), which is particularly useful for cognitive training studies (von Bastian et al., 2013). The effectiveness of this tool on cognitive interventions has been reported in previous studies (Langer et al., 2013; von Bastian and Eschen, 2016). We included two subtests “Complex span” and “Tower of Fame.” These tasks train storage, processing, and coordination of information, which are the key functions of working memory (Oberauer et al., 2003).

The battery “TAP” (Version 2.3.1., Zimmermann and Fimm, PsyTest) was used for training a variety of attentional aspects, such as alertness and divided attention. The completion of these tasks requires well-working memory functions, such as storage and processing of information. Moreover, TATOOL uses an adaptive training algorithm, and this means that the level of difficulty of the tasks was increasing based to the performance of the participant (von Bastian et al., 2013). Executive functions, memory, and processing speed were trained using a computer-based cognitive training program provided by “Cogpack” (Marker Software, Ladenburg, Germany), which is an effective tool for interventions (Gates et al., 2011; Lampit et al., 2014), and we included the subtests Comparison, Searching, Logic, Anagrams, Complete a Logical Block, and Remembering.

#### Neuropsychological Assessment

For the current study, we selected the working memory tasks from the full neuropsychological battery (Wolf et al., 2018). According to the Working Memory Model (Baddeley, 1992), the subcomponents of phonological loop and visuospatial sketchpad are implicated with the memory span procedure, and their functions include the maintenance of acoustic/speech based and visually presented material information, respectively. Moreover, previous findings (Owen et al., 2005; Donolato et al., 2017) suggest an activation of DLPFC by the backward memory span tasks. For these reasons, we used the cognitive scores of verbal and visual working memory backward spans for data analysis.

A subtest of the Wechsler Memory Scale-Revised (WMS-R; Wechsler, 1987) that measures verbal short-term and working memory (digit span) was applied. Subjects were read a sequence of numbers of increasing length and were asked to repeat the sequence in the same order (forward span) or in reverse order (backward span). For the assessment of visuospatial short-term and working memory, we used a subtest from WMS-R (block span). Subjects had to tap a sequence of blocks shown by the examiner in the same order (forward span) or in reverse order (backward span).

#### MR Data Acquisition

Subjects were scanned using two different 3T-MRI scanners. A Siemens Magnetom Verio Scanner was used at the University Medical Center in Rostock and a Siemens Magnetom TrioTim Scanner at the University Medical Center in Mainz (Siemens Medical Systems, Erlangen, Germany). All participants were asked to hold as still as possible for 1.5 h. Anatomical scans were captured using a T1-weighted Magnetization Prepared Rapid Gradient Echo (MPRAGE) sequence with the following parameters: sagittal slices = 176, scan time = 4.18 min, repetition time (TR) = 1,900 ms, echo time (TE) = 2.45 ms, flip angle = 9°, field of view (FOV) = 250 mm, and voxel volumes = 1.0 × 1.0 × 1.0 mm. During the resting-state functional MRI (rs-fMRI) examination, participants were instructed to keep their eyes closed without thinking of anything in particular or falling asleep. T2-weighted scans were captured with the following parameters: scan time = 11.02 min, transversal slices = 60, slice thickness = 2.5 mm, TR = 1,056 ms, TE = 30.6 ms, flip angle = 56°, and FOV = 210 mm.

#### MR Data Pre-processing

The 60 rs-fMRIs were pre-processed by the program Data Processing Assistant for Resting-State fMRI (DPARSFA; Chao-Gan and Yu-Feng, 2010) implemented in MATLAB (MATLAB, 2016). After the removal of the first six images, we applied a series of steps including slice timing correction and realignment to eliminate the influence of head motion. All scans were checked for excessive head motion, and participants did not show head motion more than 3 mm. The realigned images were segmented into gray matter (GM), white matter (WM), and cerebrospinal fluid (CSF), spatially normalized to Montreal Neurological Institute (MNI) space using Diffeomorphic Anatomical Registration Through Exponentiated Lie Algebra (DARTEL; Ashburner, 2007), and resampled to 3 × 3 × 3 mm voxels. To reduce the influence of noise, we regressed out linear trend, 12 motion parameters, WM, CSF, and global signal as nuisance regressors. Later, the functional images were filtered with a bandpass filter between 0.1 and 0.01 Hz and smoothed with a 6-mm Gaussian kernel.

#### Definition of Region of Interest and Global FC Analysis

The DLPFC is not an anatomical structure, but rather a functional region and is involved in a variety of cognitive processes (Yarkoni et al., 2011). According to the previous studies (Smith and Jonides, 1999; Cieslik et al., 2013), there is an activation of the right hemisphere during visual working memory tasks and a bihemispheric activation for verbal working memory tasks. Cieslik et al. (2013) investigated the functional role of DLPFC in four different experiments, and they demonstrated that the right DLPFC can be subdivided in two subregions. An anterior-ventral region with increased connectivity with anterior cingulate cortex that is associated with attention and action inhibition processes, and a posterior-dorsal region with increased connectivity with the bilateral intraparietal sulci that is related to the working memory and action execution. Moreover, Owen et al. (2005) demonstrated in their meta-analysis of functional neuroimaging studies that seeds of left DLPFC were implicated in *n*-back working memory tasks. For these reasons, we used seeds of the right hemisphere (coordinates of anterior-ventral and posterior-dorsal regions in the MNI stereotactic space) (Cieslik et al., 2013) for the analysis of the visual block span backward task and for the digit span backward task seeds of the right and left hemisphere (coordinates in Talairach space according to Owen et al., 2005) (see Table 1). The Talairach coordinates were converted to MNI space for the purpose of the analysis.

**Table 1:** Coordinates of seed regions.

		***x***	***y***	***z***
**Hemispheres**				
Right	ROI 1	37	33	32
	ROI 2	30	43	23
Left	ROI 3	−37	45	21
	ROI 4	−46	19	22

*Stereotactic coordinates in Montreal Neurological Institute (MNI) space*.

**Figure 2:**
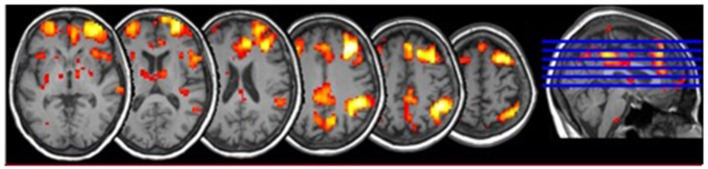
Functional connectivity of dorsolateral prefrontal cortex within the executive network including Gyrus parietalis inf., Gyrus supramarginalis, Gyrus temporalis med., Gyrus frontalis med., Gyrus cinguli.

The pre-processed functional images were used for the FC analysis based on a script from a previous study (Cole et al., 2012). We compared each GM voxel's signal time series with those of the seed regions, using Pearson's correlation coefficient. Subsequently, the positive correlations (*r* > 0) were transformed to the Fisher *Z* values, thresholded at z > 0.13 and averaged to produce a global FC value. We used positive correlations because they indicate higher connectivity strength (Cole et al., 2012). The FC masks were created for every region of interest (ROI) using DPARSFA including voxels in 4-mm radius sphere. The FC values of these voxels were computed giving the final correlation coefficient (Figure 2).

### Statistical Analysis

#### Definition of Short- and Long-Term Training Gains

The primary endpoint of this study was the prediction of the maintenance and generalization of training gains to untrained tasks. In this study, we measured the short-term and long-term maintenance of training gains based on three time points of neuropsychological assessments. We classified the participants in a subgroup that had short- and long-term training gains (ST+ and LT+) and in a subgroup that did not have short- and long-training gains (ST– and LT–). The successful short-term maintenance of training gains was defined as a performance improvement from pre-training to post-training assessment (posttest–pretest of trained subject > mean difference score of control group). To avoid test–retest effect, differences in scores between the assessments had to be greater in the experimental group than in the control group. This means that, subjects in the experimental group, who had a greater difference score than the mean difference of the control group, were classified to the subgroup who had training gain. Subsequently, we measured the long-term maintenance of training gains. A successful long-term maintenance was defined as the performance improvement from posttest to follow-up and maintenance of improvement (follow-up–posttest ≥ mean difference score of control group). Differences in scores between the assessments had to be greater in the experimental group than in the control group.

#### Effects of Cognitive Training

We used R (R Core Team, 2018) and ggeffects (Lüdecke, 2018) to perform mixed effects analysis to assess the effect of cognitive training on the cognitive performance of the experimental group. We measured the cognitive scores at 3 time points to assess the differences in performance between experimental and control group directly after the training and 12 weeks later.

#### Prediction of Successful Maintenance of Training Gains

Logistic regression models were built to assess the likelihood of successful maintenance of gains after the cognitive training when the FC increased using age, sex, education, baseline performance, center, and FC of four different ROIs of DLPFC as predictors. For each ROI, we built a separate model.

#### Prediction of Performance Improvement Across Time

We used R (R Core Team, 2018) and the package lme4 (Bates et al., 2015) to perform linear mixed effects analysis of relationship between the change of cognitive performance across time and the global FC of ROIs in DLPFC, age, sex, education, baseline performance, time of measurement, and center. Multilevel modeling has the advantage of fitting a growth model for each subject, where repeated cognitive measurements are nested within every person (Langer, 2009). We fitted a mixed effect model with the cognitive scores of visual span backward task as outcome variable, the covariates age, sex, education, center, and the interaction between FC of ROIs and time of measurement as fixed effects, and intercepts for subjects as random effects. Subsequently, we fitted a mixed effect model using the cognitive scores of digit span backward task as outcome and the aforementioned covariates as fixed and random effects.

## Results

### Descriptive Statistics

Demographic characteristics of total sample size and separately by recruiting centers for experimental and control groups are listed in Table 2. Demographic characteristics of the subgroups (ST+/ST–, LT+/LT–) are listed in Tables 3, 4. The results demonstrated no significant differences between the groups.

**Table 2:** Demographic characteristics for the experimental and control groups.

	**Center Mainz**			**Center Rostock**			**Total sample**		
	**Experimental**	**Control**	***P-*Value**	**Experimental**	**Control**	***P-*Value**	**Experimental**	**Control**	***P-*Value**
	***N =* 26**	***N =* 8**		***N =* 34**	***N =* 11**		***N =* 60**	***N =* 19**	
**Sex**			0.70^a^			0.95^a^			0.77^a^
Male	11	4		12	4		23	11	
Female	15	4		22	7		37	8	
**Age**	69.39 ± 6.03	70.2 ± 7.35	0.78^a^	68.61 ± 7.07	67.54 ± 6.34	0.77^a^	68.95 ± 6.59	68.68 ± 6.72	0.87^a^
**Education years**	15.27 ± 3.02	14.9 ± 3.8	0.97^a^	15.68 ± 1.60	14.54 ± 2.59	0.28^a^	15.5 ± 2.31	14.68 ± 3.03	0.39^a^
**IQ**	120.19 ± 9.43	120.70 ± 13.8	0.90^b^	113.25 ± 9.54	116.79 ± 9.30	0.29^b^	116.25 ± 10.02	118.43 ± 11.30	0.43^b^

a *Mann–Whitney test*.

b *t-test for independent samples*.

**Table 3:** Descriptive statistics for ST+, LT+, ST–, and LT– for visual span.

	**Short-term gains**			**Long-term gains**		
	**ST+**	**ST–**	***P-*Value**	**LT+**	**LT–**	***P-*Value**
**Training Gains of** **≪Visual Span≫**						
*N*	20	40		11	49	
**Sex**			0.13^a^			0.13^a^
Male	5	18		2	21	
Female	15	22		9	28	
**Age**	67.30 ± 5.51	69.77 ± 6.98	0.25^a^	66.54 ± 6.18	69.49 ± 6.62	0.14^a^
**Education years**	15.80 ± 1.93	15.35 ± 2.48	0.78^a^	15.81 ± 1.66	15.42 ±2.44	0.77^a^
**IQ**	117.23 ± 9.18	115.76 ± 10.50	0.57^b^	120.30 ± 8.41	115.34 ± 10.21	0.10^b^

a *Mann–Whitney test*.

b *t-test for independent samples*.

**Table 4:** Descriptive statistics for ST+, LT+, ST–, and LT– for digit span.

	**Short-term gains**			**Long-term gains**		
	**ST+**	**ST–**	***P-*Value**	**LT+**	**LT–**	***P-*Value**
**Training Gains of** **≪Digit Span≫**						
*N*	25	35		4	56	
**Sex**			0.19^a^			0.62^a^
Male	12	11		2	21	
Female	13	24		2	35	
**Age**	67.52 ± 5.57	69.97 ± 7.13	0.22^a^	70.25 ± 3.86	68.85 ± 6.75	0.32^a^
**Education years**	15.72 ± 2.28	15.34 ± 2.35	0.45^a^	16.75 ± 1.25	15.41 ±2.34	0.20^a^
**IQ**	115.06 ± 10.24	117.10 ± 9.93	0.44^a^	116.40 ± 12.39	116.24 ± 9.97	0.83^b^

a *Mann–Whitney test*.

b *t-test for independent samples*.

### Effects of Cognitive Training

**Figure 3:**
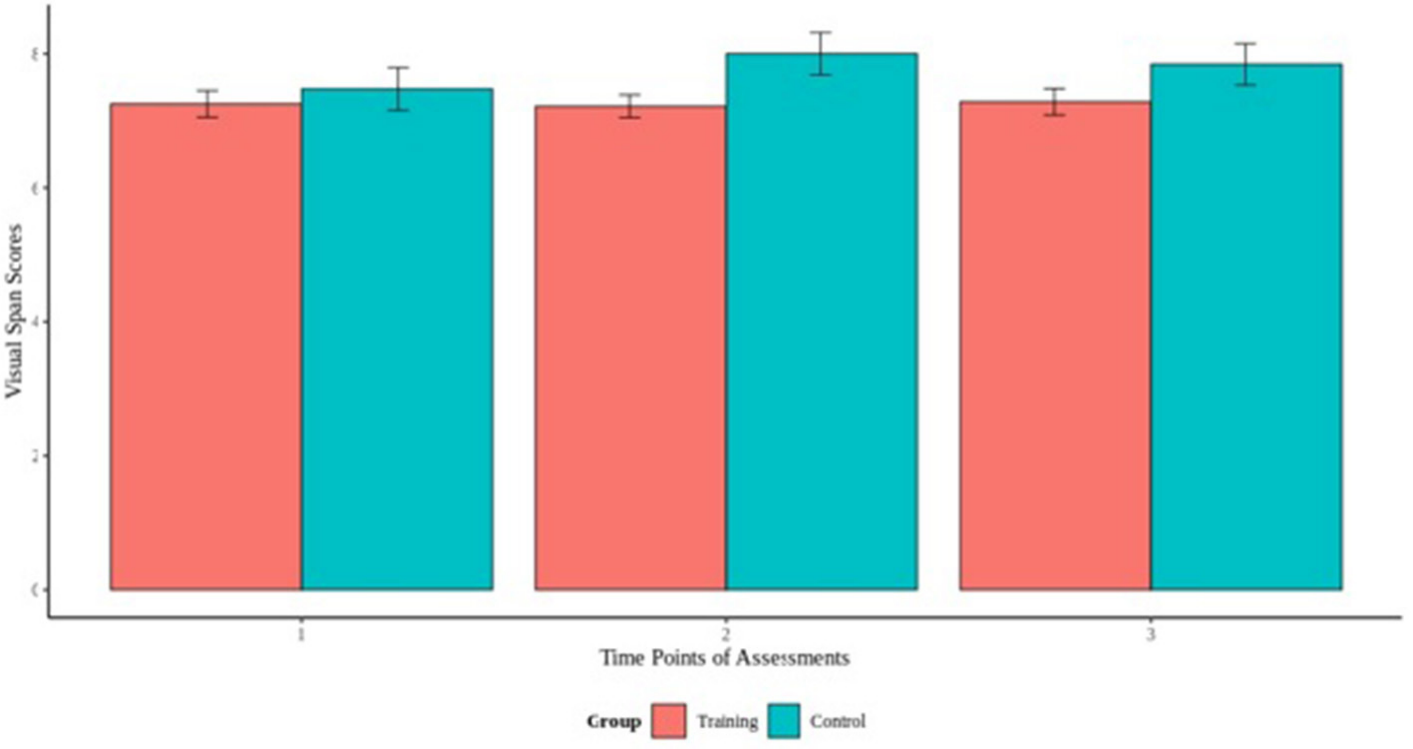
Visual span backwards scores across the 3 time points of assessments.

**Figure 4:**
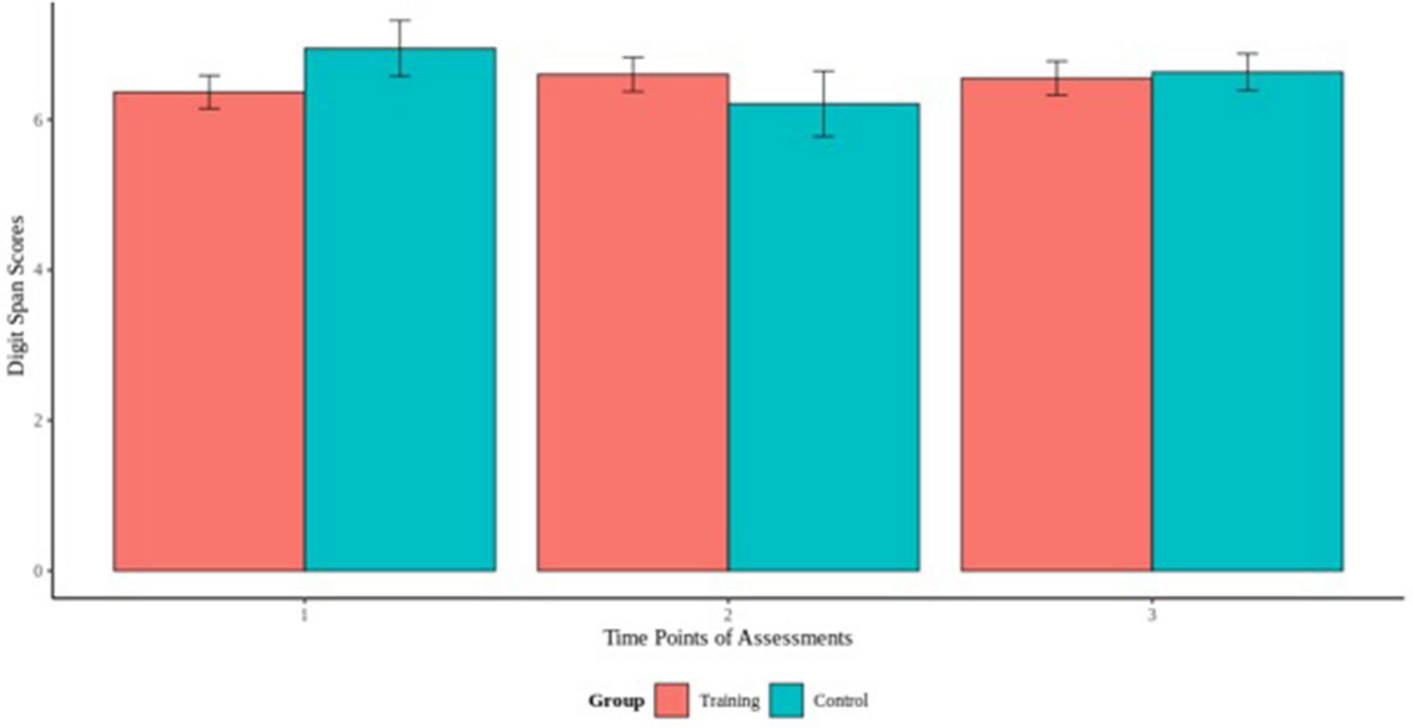
Digit span backwards scores across the 3 time points of assessments.

To assess the effect of cognitive training on the cognitive performance on the visual and digit span backward tasks, we fitted a mixed effect models using the cognitive scores of the experimental and control as outcome and the interaction term between group and time of measurement as fixed effect. Our results did not reveal any significant group by time interaction for visual span task, indicating that the experimental group did not differ from the control group, and there was no effect of the cognitive training across time (Figure 3). For the digit span task, our model revealed a group by time interaction [*B* = −0.25, *t*_(156)_ = −1.26, *p* > 0.05] that indicated differences between the groups (Figure 4). Specifically, additional analyses showed a significant group by time interaction between the first and second cognitive measurements of the experimental group [*B* = −0.98, *t*_(77)_ = −2.53, *p* < 0.05], indicating an effect of the cognitive training.

### Association of FC With Training Gains

#### Visual Span Backward

Our results demonstrated that a high FC of the posterior-dorsal region in the right hemisphere (*x* = 37, *y* = 33, and *z* = 32) raised the likelihood for a successful long-term maintenance of gains after the cognitive training (OR = 1.873e+11, 95% CI 5.833|6.015e+21, *p* < 0.05). Similarly, subjects with high FC of the anterior-ventral region in the right hemisphere (*x* = 30, *y* = 43, and *z* = 23) were more likely to exhibit a successful long-term maintenance of training gains (OR = 1.846e+10, 95% CI 7.906|4.312e+19, *p* < 0.05). The logistic models for short-term training gains did not reveal any significant effect for FC, but they indicated that a high baseline performance decreased the relative likelihood for short-term training gains.

#### Digit Span Backward

The binary logistic models did not demonstrate any significant influence of regional FC on the successful short- and long-term maintenance of training gains. We found that age had an influence on the prediction of the successful short-term maintenance of gains, indicating that younger participants were more likely to have short-term training gains. Sex was also a significant predictor of short-term training gains, indicating that males were more likely to maintain training gains than females. Moreover, our results revealed that participants with high baseline performance had a decreased likelihood for the successful short-term maintenance of training gains. Results from the logistic regression models for each ROI are reported in the Supplementary Table 1.

#### Association of FC With Cognitive Change After Training

##### Visual Span Backward

We found a significant interaction between the FC of the anterior-ventral region in the right hemisphere (*x* = 30, *y* = 43, and *z* = 23) and time of measurement [*B* = 6.09, *t*_(118)_ = 2.54, *p* < 0.05], indicating that a higher FC was associated with better performance over time. Additionally, the model showed a significant effect of sex, indicating that males had better performance over time than females [*B* = 0.77, *t*_(55)_ = 2.55, *p* < 0.05].

##### Digit Span Backward

Our models did not reveal any significant influence of FC on cognitive change. The covariate of sex was significant in our models [*B* = 0.99, *t*_(55)_ = 2.55, *p* < 0.05], indicating better performance of males than females across the three times of measurements.

## Discussion

In a hypothesis-driven approach, we determined the FC in *a priori* selected key regions of DLPFC, previously associated with working memory performance, to predict training gains and cognitive change in older people undergoing multimodal cognitive training. The DLPFC is believed to be involved in the working memory processes (Yarkoni et al., 2011; Cieslik et al., 2013), and several studies suggest that a high level of FC of DLPFC is associated with increased cognitive performance (Takeuchi et al., 2013; Chapman et al., 2015; Cao et al., 2016). To address this issue, we used rs-fMRI scans to investigate, whether a high FC of the anterior-ventral and posterior-dorsal regions of DLPFC was associated with the performance improvement and successful maintenance of cognitive gains after training.

Our results demonstrated that a higher FC of anterior and posterior region of the right hemisphere was a significant predictor of successful long-term maintenance of gains of the visual span backward task. This confirms previous findings (Cieslik et al., 2013), suggesting that during visual memory tasks there is an activation of regions of the right hemisphere. In contrast, although studies suggest bihemispheric activation during verbal memory tasks (Owen et al., 2005; Cieslik et al., 2013), in our study a high FC of regions of both hemispheres was not a significant predictor of successful maintenance of gains for the digit span backward task. Furthermore, subjects with a higher FC of the anterior-ventral region of the right hemisphere of DLFPC had more pronounced performance improvement in the visual span backward task. We did not find a significant association of FC and performance improvement in the digit span backward task. This discrepancy may be due to the fact that we used specific seed regions of DLPFC, based on previous fMRI studies, which represent a small part of DLPFC and do not reflect the entire activation pattern of the DLPFC. In addition, we focused on DLPFC due to the relevant role with working memory processes, but we did not investigate the activation patterns of other regions which are involved in verbal working memory processes, such as the basal ganglia or cerebellum (Chang et al., 2007; Moore et al., 2013; Marvel and Desmond, 2015; Emch et al., 2019). Furthermore, the discrepancy in our results may also be due to the study limitations. A replication on a bigger sample could help us to understand the association of these regions with the verbal WM.

In this study, we aimed to investigate whether a 4-week training of multiple cognitive domains improved the performance on untrained cognitive tasks. We focused on WM because it is the most vulnerable cognitive domain in aging (Bopp and Verhaeghen, 2005). We aimed to train key functions of WM, such as storage, processing, and coordination of information (Oberauer et al., 2003) by including well-established and appropriate tools in the cognitive training (Gates et al., 2011; Langer et al., 2013; Lampit et al., 2014; von Bastian and Eschen, 2016). Our results revealed that the experimental group compared to the control group showed performance improvement on the digit span backward task and not on the visual span backward task. Specifically, the improvement occurred between the first neuropsychological assessment (pre-training phase) and the second directly after the training (post-training phase). The results did not meet our expectation that there would be an improvement on both of the tasks. Based on the previous studies (Mahncke et al., 2006; Schmiedek et al., 2010), cognitive training in healthy older adults could enhance the performance on cognitive tasks, even on untrained tasks within the trained cognitive domain (near transfer effect) (Kelly et al., 2014). A possible explanation for the lack of effect on visual span backward could be that spatial WM is more affected in aging than the verbal WM (Nagel et al., 2009), and the visual span backward is a task that requires manipulation of visuospatial information. Another explanation for these results could be that the duration of the overall neuropsychological assessment was ~4 h for the baseline assessment and 3 h for the subsequent assessments, and the visual span task was tested in the middle of the neuropsychological session, after the digit span task. The performance of participants could be affected because they had already performed multiple tasks and they might have felt overstrained. It is possible *post-hoc* to state that the measures may not have been sensitive enough. This would mean, however, to point out which measures would have been more sensitive. Indeed, more novel digital-based assessments using continuous monitoring of cognitive function may be more sensitive in future. Such measures are becoming available now but were not widely available when we started this trial.

Moreover, our results demonstrated limited training gains in healthy older adults. In the visual span backward task, 33.4% (20 of 60) of the subjects demonstrated successful short-term maintenance of gains and 18.4% (11 of 60) long-term. In the digit span backward task, 41.7% (25 of 60) had successful short-term maintenance of gains and only 6.7% (4 of 60) had long-term maintenance. Our findings are consistent with previous studies, indicating that the maintenance of training gains is limited in aging, but it does exist (Schmiedek et al., 2010; Wolf et al., 2014; Heinzel et al., 2016, 2017). However, our results differ previous findings (Dahlin et al., 2008; Borella et al., 2010; Brehmer et al., 2012; Zinke et al., 2014), which reported the maintenance of gains over a time period of 3–18 months after the training in older and younger adults. We demonstrated that healthy older adults were more capable to maintain the training gain directly after the training than to maintain the gain 3 months after the training (see Tables 3, 4). A meta-analysis of Kelly et al. (2014) revealed that the maintenance of gains depends on the duration of training, and more training sessions can result in longer maintenance of training gains. Although our subjects underwent 12 training sessions, the maintenance of gains was mostly restricted to the time period after the training. A possible explanation for the immediate effect could be the habituation to the procedure of neuropsychological assessment or the increased impulse to achieve high cognitive scores after the training period. Based on the temporal context of transfer of gains (Barnett and Ceci, 2002), a shorter time period between training and cognitive measurement could have a stronger impact on the maintenance of gains rather than a measurement few months after the training.

Finally, we aimed to investigate the individual factors and their role on prediction of transfer and maintenance of training gains and performance improvement. Our findings indicated that subjects with a high baseline performance on both WM tasks were less likely to have a successful maintenance of gains. This means that subjects with low baseline performance improve after cognitive training in comparison to those who already reached a peak in their performance. Our results confirm previous findings (Zinke et al., 2012, 2014), suggesting that a low baseline performance can be a predictor of transfer. A possible explanation for this result could be the use of cognitive resources in old age (Zinke et al., 2012). Older adults, who often use cognitive resources already in their everyday life, are able to achieve a high performance level and that is why they have no additional benefit from a cognitive training. In contrast, older adults using less cognitive resources at baseline may have more benefit from training because with the help of training they can activate their cognitive resources and they are challenged to achieve the best possible score. Moreover, the recruitment strategy was based on advertisement, a way that attracts people, especially with high education as they are more cognitively agile; thus, that could explain the bias of well-educated participants who had a high baseline performance and did not have a benefit from training. Age was associated with the effects of cognitive training in most studies, with younger participants showing higher transfer effects (Bürki et al., 2014; Zinke et al., 2014). Consistently, in our study, we found that younger age was significantly associated with short-term training gains for the digit span backward task. Lastly, men had more pronounced performance improvement in both of the tasks. Sex was also a significant predictor of successful short-term maintenance of training gains for the digit span backward, indicating that men were more likely to benefit from training than women. This agrees with the findings of the meta-analysis of Blume et al. (2010), which showed that men had higher likelihood to maintain training gains, albeit at a small effect size of *r* = 0.12 for sex. However, more studies are needed to investigate sex differences in training gains because intervention studies usually treat older adults as a homogenous group. Moreover, our findings are consistent with previous studies, which demonstrated a male advantage on visual and verbal working memory measures (Speck et al., 2000; Lejbak et al., 2011; Zilles et al., 2016).

Our study has several limitations. First, our sample size was relatively small, which affects the generalizability of the results. Although we demonstrated significant results in this sample, replication will be required in independent studies. Moreover, our sample included participants not only with low performance, but also high performers who could not benefit from the training. This, however, reduced the extent of intervention effects. Second, the FC analysis based on activations of specific DLPFC regions from previous fMRI studies is both a strength and a limitation of the current study. It is a strength as it reduced the risk of finding spurious results, but we did not explore associations within the entire DLPFC. Still, we think it is very valuable to test directed hypotheses with *a priori* associations. A further limitation is that the study design included only baseline fMRI and this limited the possibility of observation of training effects and longitudinal changes in FC.

## Conclusions

In the current study, we could demonstrate that transfer and maintenance of training gains occur in aging, but it remains limited. We further demonstrated that a high FC of *a priori* seed regions of DLPFC could predict a successful maintenance of training gains and performance improvement across time on a visuospatial working memory task. The fact that we demonstrated significant results even in a small sample gives us the trigger to further explore the brain regions and activation patterns. This will provide us information about the determination of training gains likelihood and the identification of healthy older adults who could benefit from training. This could potentially serve as an index for the stratification of people into those who more likely benefit from a cognitive intervention and others who might be more responsive to alternative intervention strategies.

Building on our findings, further studies could focus on the different brain regions and explore the activation patterns and the association with other cognitive domains. Moreover, far transfer effects could be investigated and help us to understand if untrained cognitive domains could be improved and how the training gains could be applied and help older people to improve their everyday life.

## Data Availability Statement

The raw data supporting the conclusions of this article will be made available by the authors, without undue reservation.

## Ethics Statement

The studies involving human participants were reviewed and approved by Mainz: Ethics Commission of the Landesärtzekammer Rheinland-Pfalz, Rostock: Ethics Commission of the Rostock University's Faculty of Medicine [Reference number: 837.385.15 (10153)]. The patients/participants provided their written informed consent to participate in this study.

## Author Contributions

SF performed analyses and interpretation of the data, drafted, and revised the manuscript. JW performed analyses and interpretation of the data. MD participated in the pre-processing and analysis of the rs-fMRI data. DW, OT, AM, and AF contributed significantly to the conception and design of the study. FF, KK, and DR contributed to the acquisition of the data. BK also contributed to the acquisition, provided feedback, and revised the manuscript. HB contributed significantly to the design of the study, provided feedback, and revised the manuscript. ST was involved in all stages of the work, contributing to the study design, research question and analyses, and critically revising the manuscript. All authors read and approved the manuscript.

## Conflict of Interest

ST has done the works as follows (all in Germany): MSD Sharp & Dohme GmbH, Lindenplatz 1, 85540 Haar; 11/09/2018: Quality circle for physicians in Kühlungsborn, Talk: “Dementia and Diabetes—current report”; 14/11/2018: MSD Expert-forum: NAB Alzheimer in Munich, participator as consultant; 13/08/2019: Event “Diabetes and Dementia” in Rostock, Talk: “Dementia and Diabetes—current report”; ROCHE Pharma AG, Emil-Barell-Str. 1, 79639 Grenzach-Wyhlen; 12/09/2019: 3. National Advisory-Board in Frankfurt (Main), participator as consultant; 27/09/2019: ROCHE Symposium at the DGN Congress in Stuttgart, Talk: “Amyloid as target for diagnosis and treatment in Alzheimer's disease”; Biogen GmbH, Riedenburger Straße 7, 81677 Munich; 9/2020: Advisory Board Biogen. The remaining authors declare that the research was conducted in the absence of any commercial or financial relationships that could be construed as potential conflicts of interest.
