# Supplementary material for: Dorsolateral Prefrontal Functional Connectivity Predicts Working Memory Training Gains
Source: Front Aging Neurosci. 2021 Mar 1;13:592261. doi: 10.3389/fnagi.2021.592261 (PMC7956962; doi:10.3389/fnagi.2021.592261)
Supplement: Supplementary file 1 [file Table_1.docx]

Supplementary Table

**Supplementary Table 1. Results from logistic models using Digit span backwards scores**

**O.R. 95% CI P value**
**ROI 1 (37/33/32)
 Sex** 0.210 0.045│0.980 <.05 **Baseline performance** 0.476 0.299│0.758 <.05
 **Age**  0.905 0.815│1.005 .06 **ROI 2 (30/43/23)**

**Baseline performance** 0.524 0.342│0.802 <.05

**ROI 3 (-37/45/21)**

0.511 0.331│0.789 <.05

**ROI 4 (-46/19/22)**

**Sex**  0.219 0.049│0.987 <.05
 **Baseline performance** 0.498 0.319│0.777 <.05**___**
